# Risk of synchronous endometrial disorders in women with endometrioid borderline tumors of the ovary

**DOI:** 10.1186/s13048-018-0405-0

**Published:** 2018-04-19

**Authors:** Shuang-zheng Jia, Jun-ji Zhang, Jun-jun Yang, Yang Xiang, Zhiyong Liang, Jin-hua Leng

**Affiliations:** 1 Department of Obstetrics and Gynecology, Peking Union Medical College Hospital, Chinese Academy of Medical Science & Peking Union Medical College, Beijing, 100730 People’s Republic of China; 2 Department of Pathology, Peking Union Medical College Hospital, Chinese Academy of Medical Science & Peking Union Medical College, Beijing, People’s Republic of China

**Keywords:** Endometrioid borderline ovarian tumor, Endometrial disorder, Fertility, Risk factor

## Abstract

**Background:**

Synchronous endometrial disorders have been poorly studied in women with endometrioid borderline ovarian tumors (EBOT). The aims of this study were to investigate the risk of endometrial disorders among women with EBOT and associated factors, as well as their oncological and fertility outcomes.

**Results:**

This retrospective study included 33 women with EBOT. Their mean age was 41.9 years, and endometria were evaluated in 25 of these patients. The prevalence of synchronous endometrial disorders was 52.0% (*n* = 13/25) and this incidence was 41.4% (*n* = 46/111) after systematic analysis. Univariable analysis showed that EBOT patients who were younger, nulliparous, and had experienced abnormal vaginal bleeding were more likely to have synchronous endometrial disorders. The median follow-up was 54 months (range: 14-250 months), and three patients (10.3%) developed recurrences. No deaths due to EBOT were recorded. Among the nine nulliparous women treated conservatively who were attempting to conceive, only one (11.1%) pregnancy resulted in a live birth.

**Conclusions:**

Synchronous endometrial disorders are common in women with EBOT, especially in those who are younger, nulliparous, and have experienced abnormal vaginal bleeding. Thus, endometrial sampling should be performed in women with EBOT undergoing conservative surgery, and a hysterectomy should be performed in cases requiring radical treatment.

## Background

Borderline ovarian tumors (BOT), indolent neoplasms characterized by the presence of cellular proliferation and nuclear atypia in the absence of destructive stromal invasion, account for 10-20% of all epithelial ovarian cancers [1, 2]. Over the past two decades, considerable work has been performed to investigate the clinicopathologic features of serous and mucinous BOTs, as well as the oncological and fertility outcomes of patients with BOTs [3–5]. However, due to its rare incidence, endometrioid BOTs (EBOTs), the third most common BOT, have been poorly studied [6]. Most studies on EBOTs have focused mainly on the pathological characteristics and included only brief descriptions of management and follow-up [6–10]. Additionally, only a few cases that were treated with conservative surgery have been reported; therefore, the fertility outcomes of these patients are unknown [6, 9].

Although approximately one-third of patients with EBOT have endometrial hyperplasia and/or carcinoma, to the best of our knowledge, no specific data are available that identify groups at high risk for EBOT [10]. Furthermore, staging surgery without endometrial biopsy remains the cornerstone for women with EBOT who hope to become pregnant. Thus, to better understand the course of EBOT and to improve the surveillance of these patients, a thorough exploration of the prevalence of synchronous endometrial disorders in women with EBOT and identification of potential risk factors is urgently needed.

The primary objective of our study was to investigate the risk of synchronous endometrial disorders among women with EBOT and to identify their risk factors. We also evaluated the oncological and fertility outcomes in these EBOT patients.

## Methods

### Patients

After obtaining approval from the Institutional Review Board at the Peking Union Medical College Hospital (approval No. S-K351), consecutive patients with EBOT who were treated or referred to our hospital were identified through a search of medical records between 1995 and 2015. Medical records, including patient charts and operative, pathological, and follow-up records, were comprehensively reviewed. All EBOT were diagnosed by two gynecologic pathologists at our institution, and the FIGO 2013 staging system for epithelial ovarian tumors was used for determining disease stage based on the operative descriptions and pathology records [11].

### Surgical management

Conservative surgery was defined as fertility-sparing, wherein the uterus and at least part of one ovary are salvaged, whereas radical surgery was defined as bilateral salpingo-oophorectomy (BSO) with or without a hysterectomy. A proper staging surgery consisted of carefully inspecting all peritoneal surfaces with peritoneal washing, random or oriented multiple biopsies, and omentectomy [1]. For those patients referred to our hospital after EBOT diagnosis, a second surgery was offered. The extent of surgery was determined by the combination of previous surgeries and surgeries performed at our hospital. Pelvic lymphadenectomy and adjuvant platinum-based chemotherapy were performed according to the surgeon’s preference.

Post-operative evaluation, including clinical examination, vaginal ultrasonography, and CA-125 measurements, was offered every 3 months during the first 2 years, every 6 months during the next 3 years, and yearly thereafter.

### Literature review

We used MEDLINE and PubMed to perform a literature search. The following search terms were used: “borderline” OR “atypical” OR “low-malignant potential” OR “low malignant potential” AND “endometrioid” AND “tumor” AND “ovary” OR “ovarian”. Only articles published in English were included.

### Statistical analysis

Primary outcomes included coexisting endometrial disorders in women with EBOT, defined as endometrial cancer, atypical hyperplasia (AH), and endometrial hyperplasia without atypia, during primary surgery and/or follow-up. Data were expressed as mean ± standard deviation, median (range), or number (percentage). For inferential analysis, an independent *t* test and Fisher’s exact test were used to compare study variables between cases (women with synchronous endometrial disorders) and controls (women with normal endometrium) when appropriate. Due to the small sample size, multivariable analyses were not performed. All data were analyzed using SPSS 23.0 and *p* < 0.05 was considered significant.

## Results

### Patient characteristics

A total of 590 BOTs were identified, of which 33 (5.6%) were diagnosed as EBOT. The mean age of the patients was 41.9 years (range: 23-81 years). More than two-thirds of the participants (69.7%) were premenopausal. Of the 16 nulliparous women, four were infertile prior to treatment of their ovarian tumor. Ovarian cysts were the most common indications for surgery (75.8%; 25/33) and elevated serum CA-125 values were found in more the half of the patients (56.7%; 17/30). The mean tumor diameter was 7.3 cm and bilateral tumors were present in 4 patients (including three recurring in the contralateral ovary). Demographic features and disease characteristics of the 33 patients are detailed in Table 1.

**Table 1:** Demographic features and disease characteristics of participants

Variable	n (%)	Mean ± SD	Median
Age (y)		41.9 ± 12.85	
Nulliparous	16 (48.48%)		
Preoperative serum CA-125 (*n* = 30)			39.9 (8-1037)
< 35 IU/L	13 (43.33%)		
> 35 IU/L	17 (56.67%)		
Tumor diameter (cm)		7.3 ± 4.56	
Surgical extent			
Conservative surgery	17 (51.52%)		
Radical	16 (48.48%)		
Staging surgery	18 (54.55%)		
Stage			
I	31^b^ (93.94%)		
II-III	2 (6.06%)		
Histological feathers			
Stromal microinvasion	7 (21.21%)		
Intraepithelial carcinoma	4 (12.12%)		
Implants	2^c^ (6.06%)		
Coexisting endometriosis	10 (30.30%)		
Endometrial evaluations	25 (75.76%)		
No lesions	12 (48.00%)		
Hyperplasia without atypia	2 (8.00%)		
EIN	5 (20.00%)		
Endometrial cancer	6 (24.00%)		
Additional treatment			
Chemotherapy	10^d^ (30.30%)		
Radiotherapy	3 (9.09%)		

*USO* unilateral salpingo-oophorectomy; *EIN* endometrial intraepithelial neoplasia

^a^Including three recurrences in the contralateral ovary

^b^Including four patients with stage IC2

^c^Including one invasive and one non-invasive implant respectively

^d^Including one melphalan chemotherapy

Approximately two-thirds of the patients were treated with laparotomy (*n* = 21), including five conversions from laparoscopy to laparotomy during a 2-step staging procedure. Among the 33 patients, 17 underwent conservative surgery and 16 underwent radical surgery after a one-step (*n* = 12) or a two-step procedure (*n* = 4). Staging surgery was performed for 18 patients, of which five were conservative. Fourteen patients underwent lymphadenectomy; no positive findings were recorded. Postoperatively, 10 women received chemotherapy and radiotherapy was prescribed to three women to treat synchronous endometrial cancer.

### Pathological findings

Histological analysis revealed that 10 patients had coexisting peritoneal or ovarian endometriosis, including six that occurred in the ipsilateral ovary. Intraepithelial carcinoma (IEC) and stromal microinvasion were found in four and seven patients, respectively; all but two patients had stage I disease, including four patients with stage IC2. One patient with EBOT and microinvasion had an invasive implant of the sigmoid colon. Noninvasive implants of the omentum were seen in a 43-year-old woman with bilateral EBOT who had positive cytology as well as right ovarian IEC associated with EBOT.

### Synchronous endometrial disorders

Twenty-five women had endometrial evaluations and 13 patients had synchronous endometrial disorders, including six women with endometrial cancer, five with atypical hyperplasia (AH), and two with endometrial hyperplasia without atypia. The crude prevalence of endometrial disorders in the women with EBOT was 52.0% (*n* = 13/25).

Analyses of the data indicated that EBOT patients who were younger, nulliparous, and had abnormal vaginal bleeding (AVB) were more likely to have synchronous endometrial disorders (*P* = 0.02; *P* = 0.03; *P* = 0.001; respectively; Table 2). However, no histological variables were found to be significantly associated with endometrial disorders.

**Table 2:** Risk factors of synchronous endometrial disorders in women with EBOT

Variables	Cases (*n* = 13)	Controls (*n* = 12)	*p*
Age (y)	39.1 ± 10.4	50.9 ± 13.1	0.02^a^
Menopausal			0.11^b^
No	10	5	
Yes	3	7	
AVB			0.001^b^
No	3	11	
Yes	10	1	
Nulliparous			0.03^b^
No	5	10	
Yes	8	2	
Tumor diameter	5.7 ± 2.1	8.2 ± 4.3	0.07^a^
CA-125			0.25^b^
< 35 IU/L	6	3	
> 35 IU/L	5	7	
Coexisting endometriosis			0.45^b^
No	10	8	
Yes	3	4	
Stage			0.28^b^
I-IC1	11	8	
IC2-III	2	4	
IEC			0.47^b^
No	12	10	
Yes	1	2	
Microinvasion			0.46^b^
No	11	9	
Yes	2	3	

^a^Independent sample *t* test assuming equal variance

^b^Fisher’s Exact test

### Oncological and fertility outcomes

Four patients were lost to follow-up after surgery. Among the remaining 29 patients, the median follow-up was 54 months (range: 14-250 months). Three patients (10.3%) developed recurrences but no women died of their disease. The time to recurrence among these 3 patients was 18 months, 100 months, and 180 months, respectively. These three women were young (23, 30, and 30 years old, respectively), nulliparous, and had co-existing endometrial disorders (two women with AH and one women with EC). Conservative surgeries without staging were performed at their initial treatment. Two women recurred on the residual contralateral ovary and one woman relapsed as bilateral recurrence (Table 3).

**Table 3:** Details of the three patients who experienced recurrence

Age	Initial surgery	Initial pathology	Time to recurrence	Secondary surgery	Secondary pathology	Coexisting endometrial disorders
30	Lap UC+Hys	EBOT	18 months	Lap BUC+D&C	Bilateral EBOT	EC
30	LSO+LN	EBOT	240 months	Lap RSO	EBOT	EIN II-III
23	RSO	EBOT	100 months	Lap UC	EBOT	EIN II-III

*Lap* laparoscopy; *Hys* hysteroscopy; *D&C* dilation & curettage; *UC* unilateral cystectomy; *EC* endometrial cancer; *LSO* left salpingo-oophorectomy; *RSO* right salpingo-oophorectomy; *EIN* endometrial intraepithelial neoplasia

Among the 16 nulliparous women, 13 women were treated conservatively with a fertility-sparing procedure. One patient was lost to follow-up and three women had not attempted to conceive at the time of analysis. Of the nine patients who had attempted to conceive, only one (11.1%) pregnancy resulted in a live birth. Moreover, of these nine women, five had synchronous endometrial disorders and two experienced contralateral EBOT recurrence.

### Literature review

**Fig. 1:**
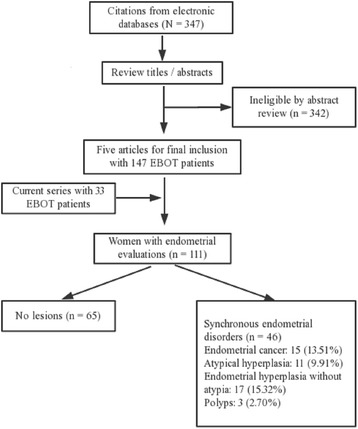
Literature review of studies of women with EBOT

We identified 347 citations from 1981 to November 2015. After reviewing titles and abstracts, only five papers that described 147 patients were included in the final analysis. Of these 147 women, 86 were evaluated for endometrial status. Thirty-three of these women were diagnosed with synchronous endometrial disorders, including nine who had endometrial cancer. Together with the present study, a total of 111 women with EBOT underwent endometria sampling and 46 were diagnosed with endometrial disorders, indicating a crude prevalence of 41.4% (Fig. 1).

## Discussion

EBOT is a rare tumor that represents 0.2% of all epithelial ovarian tumors. To our knowledge, this is the first study to evaluate risk factors for synchronous endometrial disorders in women with EBOT. Our results indicated that synchronous endometrial disorders are highly prevalent in women with EBOT, especially in those who are younger, nulliparous, and have AVB. Our study also demonstrated that, although women with EBOT have a favorable prognosis, their fertility outcomes are poor.

When treating EBOT patients, physicians must decide whether to include endometrial curettage as a therapeutic approach. Although few studies have evaluated the incidence of endometrial disorders in women with EBOT, studies by Bell and Kurman and Snyder et al. found that 12.5% (3/24) and 68.4% (13/19), respectively, of EBOT patients also had endometrial disorders [7, 9]. However, our study revealed that the prevalence of synchronous endometrial disorders was 52%; this incidence remained high (41.4%) following systematic analysis. Thus, as recommended by Uzan et al., endometrial sampling should be performed in women with EBOT undergoing conservative surgery; in addition, a hysterectomy should be performed in cases requiring radical treatment [6, 12].

The mechanisms underlying endometrial disorders in EBOT patients remain unclear. Although one study indicated that stromal luteinizations were more common in EBOT patients with concurrent endometrial disorders, to our knowledge, no other study has confirmed this result [8]. Analyses of our data showed that EBOT patients who were younger, nulliparous, and had AVB were more likely to experience endometrial disorders. To our knowledge, this is the first study to demonstrate this relationship.

Endometrioid carcinomas of the ovary tend to coexist with various forms of endometrial neoplasia, whereas mutations in several genes, including β-catenin and PTEN, have been demonstrated to occur concurrently with endometrial cancers and endometrioid ovarian cancers [13]. Furthermore, distinct clinical characteristics, including younger age, obesity, premenopausal status, and nulliparity were demonstrated in women with synchronous primary cancers of the endometrium and ovary [14].

The finding that women with AVB were more likely to have concurrent endometrial disorders was not surprising. Endometrial cancer or atypical hyperplasia occurs in 1.31% of premenopausal women with AVB, and this incidence can increase to 17.3% in menopausal women [15, 16]. However, 88.9% (*n* = 10/11) of women with EBOT and AVB also had endometrial disorders, and this incidence was significantly higher than that of the general population. Furthermore, uterine endometrioid adenocarcinomas can also be found in women who do not show uterine symptoms [6]. More than 60% (8/13) of our study participants were nulliparous, which is consistent with a study by Eifel et al., wherein 50% of the study participants who had synchronous endometrioid/endometrioid tumors were nulliparous [17]. Similarly, Herrinton et al. reported a lower than expected mean parity in women with synchronous endometrial and ovarian cancers [18].

In our study population, women with EBOT and synchronous endometrial disorders were approximately 12 years younger than those with EBOT alone. Similar findings were also reported in women with coexisting endometrial and ovarian carcinomas [19]. The combination of younger age, AVB, and nulliparity, as well as other characteristics, including obesity, in women with synchronous endometrioid/endometrial carcinomas suggests the involvement of a hormonal “field effect” in the development of these simultaneous tumors, particularly endometrioid cell-type tumors [14, 19]. In recent years, accumulating evidence has suggested that ovarian cancer arises from the pleiotropic interactions of the committed stem cells within the ovary, the surrounding microenvironment, and the infiltrating immune cells [20, 21]. Thus, whether these interactions may contribute to the development of synchronous endometrial and ovarian disorders needs further exploration.

With respect to oncological outcomes, over the past decade several studies have demonstrated the safety of fertility-sparing surgery in early stage epithelial ovarian cancer patients whose preservation of reproductive potential is pivotal to their quality of life [22–24]. Previous studies have found that women with EBOT have a favorable prognosis, with minimal recurrence and no EBOT-associated deaths [6–9]. However, in this study, three patients experienced recurrences, of which two recurred on the contralateral ovary and one relapsed as a bilateral recurrence. All three women were young (23, 30, and 30 years old, respectively), nulliparous, and had synchronous endometrial disorders. Uzan et al. reported a malignant transformation, wherein a 37-year old woman suffered an invasive relapse as endometrioid carcinoma after radical treatment for her first recurrence [6]. This unusual case, in combination with our three recurrences, emphasizes the importance of endometrial biopsy in women with EBOT and the need for an intensive post-operative follow-up, especially in younger patients.

Promising data have been reported for fertility outcomes in women with serous/mucinous BOTs after conservative management. One study reported a pooled estimated spontaneous pregnancy rate of 54% that increased to 80% after fertility treatment [4]. In women with advanced-stage serous BOTs, a long-term follow-up study reported a pregnancy rate of 57.1% [25]. For BOTs, conservative treatment, patient age, and BOT histologic subtype have been reported to influence fertility outcomes; however, for patients with EBOT, there is limited information about fertility outcomes [26–28].

In this study, we demonstrated for the first time that women with EBOT have poor fertility outcomes. Of the nine women who attempted conception, only one (11.1%) pregnancy was achieved that resulted in a live birth. We should note that five of these nine women developed endometrial disorders after their initial EBOT surgery, including two women with endometrial cancer. Only two women experienced AVB but endometrial sampling was not performed during the initial surgery (unpublished data). Thus, whether nulliparity itself contributes to the development of endometrial disorders or vice versa awaits further study. In addition, adherence and alterations in ovarian function following surgery could also contribute to poor fertility outcomes.

This study has several limitations. First, the present study is inherently limited by its retrospective design and small sample size; in addition, the data were collected from a single institution. As a result, the data in this study did not include all factors that could contribute to the etiology of EBOT tumors, such as body mass index. Women with obesity are at increased risk of endometrial disorders due to excess peripheral conversion of androstenedione to estrone in adipose tissue. However, to the best of our knowledge, due the rare incidence of this condition, this is the largest study to focus on endometrial sampling in women with EBOT. Second, pathological review of the specimens was not performed. The Peking Union Medical College Hospital is a major referral center and has the best gynecology and pathology department in the country. In addition, two gynecological pathologists reviewed original pathology reports. Finally, not all women included in this study underwent endometrial biopsies during their initial surgery (endometrial disorders were observed in six women during follow-up), which may have resulted in overestimation of the incidence of synchronous endometrial disorders. However, this incidence of was 41.4% following after systematic analysis.

## Conclusions

In summary, the present study demonstrated that endometrial disorders are common in women with EBOT, especially in those who are younger, nulliparous, and have AVB. Thus, endometrial sampling should be performed in women with EBOT undergoing conservative surgery, and a hysterectomy should be performed in cases requiring radical treatment. Although there were no negative oncological outcomes, the fertility rates for these patients were poor, implying that a more active approach should be taken for EBOT patients who wish to become pregnant.

## Abbreviations

AH: Atypical hyperplasia
AVB: Abnormal vaginal bleeding
BOT: Borderline ovarian tumors
BSO: Bilateral salpingo-oophorectomy
EBOT: Endometrioid borderline ovarian tumors
IEC: Intraepithelial carcinoma
